# Gut microbial changes in a specialist blister beetle larvae and their nutritional metabolic characteristics

**DOI:** 10.1002/ece3.70184

**Published:** 2024-08-22

**Authors:** Jinnan Ma, Zhaohui Fu, Xin Yang, Wenqin Ming, Xuhao Song, Chao Du

**Affiliations:** ^1^ Yunnan Normal University Kunming China; ^2^ Baotou Teachers' College Baotou China; ^3^ Key Laboratory of Southwest China Wildlife Resources Conservation (Ministry of Education) China West Normal University Nanchong China

**Keywords:** 16S rDNA amplicon sequencing, blister beetles, gut microbiota, *Hycleus cichorii*

## Abstract

Insect gut microbiota and their metabolites play a significant role in the shaping of hosts' diets and feeding habits. We conducted 16S rDNA amplicon sequencing on the gut microbiota of specialist blister beetle larvae that feed on locust eggs and artificial food at different instars, to explore the relationship between gut microbiota and the specialized feeding habit of the blister beetle larvae. There is no significant difference in the gut microbial structure among the second to the fourth instar larvae under the same rearing conditions, but the gut microbial structure of the first instar larvae was significantly different from the second to the fourth instar larvae fed by different diets. Bacteria associated with polysaccharide utilization are relatively barren in first instar larvae. Compared to the carbohydrate content between the artificial diet and locust eggs, we speculate that an excessive amount of polysaccharides in the artificial diet may be detrimental to the growth and development of first instar larvae. Gut microbiota of the second to the fourth instar larvae fed with different diets significantly differ in microbial community structure. The different bacteria, especially the metabolism‐related intestinal bacteria in locust eggs‐fed larvae, may help the hosts adapt to the environment and contribute to the production of active ingredients. The relative abundance of polysaccharide utilization‐related bacteria was significantly higher in the artificial diet‐fed larvae compared to the locust eggs‐fed larvae, which showed the same result when compared to the first instar larvae. Changes in gut microbes of blister beetle larvae and their metabolic inferences could enrich our understanding of the nutritional requirements of the specialist and help optimize the artificial diet of medicinal cantharides.

## Graphical abstract

This study represents a first attempt to uncover how changes in gut microbiota play a role in the dietary preferences and feeding habits of *Hycleus cichorii* larvae. The excessive amount of polysaccharides in the artificial diet may be detrimental to the growth and development of first instar larvae. The differences in microbial community structure between the second to the fourth instar larvae under the different rearing conditions would help the hosts adapt to the different nutritional environments and provide reference for optimization of artificial diet for beetles.

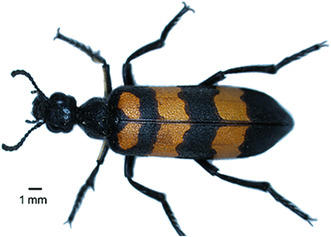

## 1 INTRODUCTION

Blister beetles are a group of insects in the family Meloidae of the order Coleoptera. Blister beetles have been utilized in traditional Chinese medicine for over two millennia, with documented medicinal applications also found in Western historical records. The medicinal component of blister beetles mainly comes from a toxic secondary metabolite called cantharidin (C_10_H_12_O_4_) (Borror et al., 1989; Evans & Hogue, 2006). Modern medical research has shown that cantharidin has significant therapeutic effects on skin diseases, chronic diseases, leukemia, and other conditions (Moed et al., 2001; Sun et al., 2016), especially for the treatment of primary liver cancer, lung cancer, breast cancer, and skin cancer (Hsia et al., 2016; Kadioglu et al., 2014; Kim et al., 2013; Le et al., 2016; Li et al., 2017).

With the extensive use of pesticides and the excessive exploitation of medicinal resources, the wild population of blister beetles has decreased. The chemical synthesis of cantharidin is difficult due to different problems such as harsh reaction conditions, complex operations, and low yield (Tan et al., 2018; Tan & Liu, 2022). Besides, the synthesis and transfer of cantharidin in blister beetles are largely unknown (Zhou et al., 2024). The current raw materials for cantharidin drugs still rely on limited wild resources, which greatly restricts the development and utilization of blister beetle medicinal resources.

Blister beetles are hypermetamorphic insects and have an extremely complex life cycle (Shintani et al., 2017). Adult beetles mainly feed on flowers and leaves of plants in the legume, nightshade, and gourd families, while the larvae must feed specifically on locust eggs or parasitize inside beehives (Bologna & Pinto, 2010; Saul‐Gershenz et al., 2018). Meloid species with a long history of medicinal use and higher cantharidin content mostly belong to the Mylabrini, Lyttini, and Epicautini tribes, whose larvae exclusively feed on locust eggs (Deyrup et al., 2021). The specialized feeding of blister beetle larvae on locust eggs is a major obstacle in the large‐scale breeding of blister beetles, which has not yielded the desired results (Fu et al., 2023).

**FIGURE 1:**
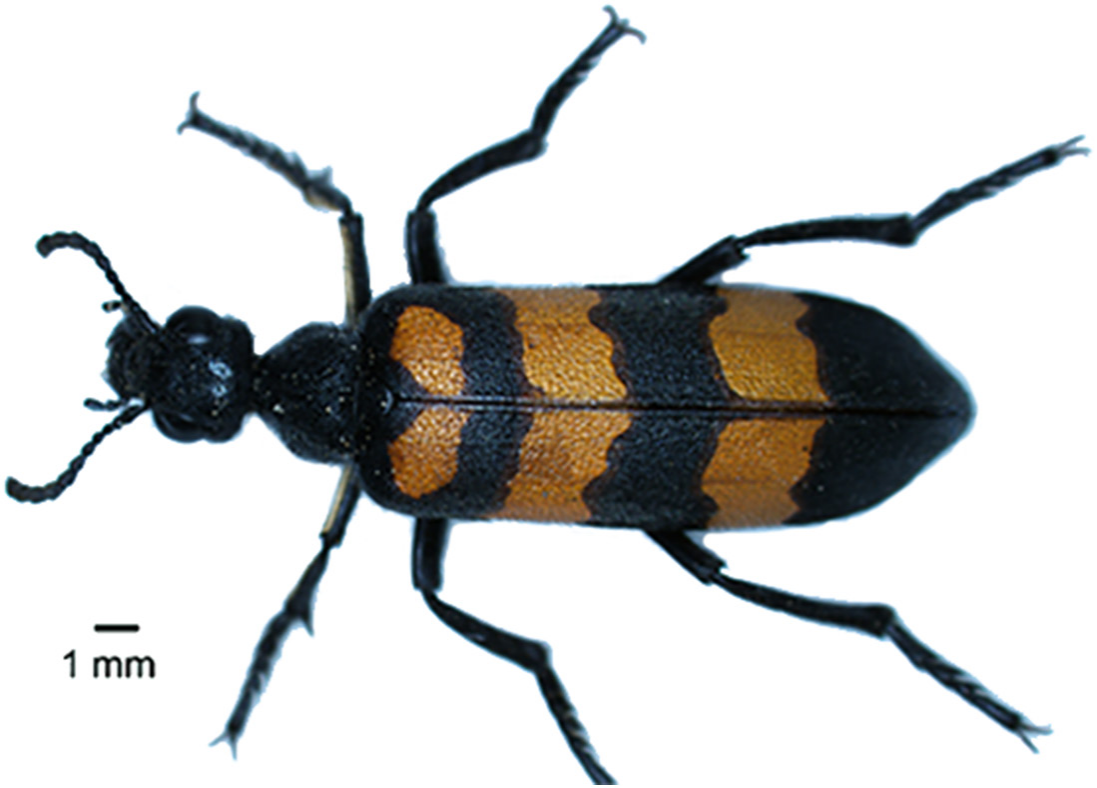
Blister beetle, *Hycleus cichorii*.

*Hycleus cichorii* Linnaeus is commonly known as the “yellow and black small blister beetle,” which is a species of blister beetle included in the Chinese Pharmacopeia (Figure 1). We have been researching the artificial breeding of *H. cichorii* larvae and found that their specialized feeding on locust eggs decreases with instar. First instar larvae can only feed on locust eggs and cannot develop under artificial feeding conditions. Many studies have shown that the dietary preferences and feeding habits of animals are not only determined by the species themselves but are also closely related to their gut microbiota (Greene et al., 2020; Heys et al., 2021; Vilanova et al., 2016). Animals with specialized dietary habits have developed a relatively stable symbiotic relationship with their gut microbiota through long‐term coevolution (Ceja‐Navarro et al., 2019; Scully et al., 2014; Zhu et al., 2011). Insects of different species with the same dietary habits had similar gut bacterial communities, whereas significant differences were noted in the composition and function of gut microbial communities in the same species of insects with different dietary habits (Zheng, 2023). Therefore, we speculate that changes in the diet of *H. cichorii* larvae during their growth process may be related to changes in their gut microbiota.

We aim to explore the impact of intestinal microbes on the specialized feeding habit of *H. cichorii*, which is a species of blister beetles included in the Chinese Pharmacopeia. We conducted 16S rDNA amplicon sequencing on the gut microbiota of larvae that feed on locust eggs and artificial food at different instars, to explore the relationship between gut microbiota and the specialized feeding of the blister beetle larvae. The results would enrich the coevolution theory of insects and their endosymbiotic bacteria, and guide the optimization of artificial diet and rearing technology of cantharides.

## 2 MATERIALS AND METHODS

### 2.1 Insect sources and rearing

Specimens of *H. cichorii* were collected at different developmental stages (larvae) to determine their gut microbiota. *H. cichorii* and the migratory locusts were reared in the laboratory of Baotou Teachers' College under the condition of 29 ± 1°C, 60% ± 5% relative humidity, and 16L:8D photoperiod. Both were continuously reared for at least three generations in the laboratory. The locusts' egg pods were collected to feed the *H. cichorii* larvae. The *H. cichorii* larvae were reared individually in 10 mL centrifuge tubes, as described by Fu et al. (2023). The first instar larvae (L1) had just hatched and were not fed by the authors. The second to fourth instar larvae were separated into seven groups according to their instar and assigned diets. Larvae of LL2, LL3, and LL4 groups were fed on locust eggs and sampled in the second, third, and fourth instar stages, respectively. Larvae of AL2, AL3, and AL4 groups were fed on the artificial diet and sampled in the second, third, and fourth instar stages, respectively. The artificial diets for the AL2~AL4 group were renewed every 5 days. The components of the artificial diet were chicken eggs, honeybees, milk powder, yeast powder, sorbic acid, honey, and water. The ingredients of the artificial diet were sterilized before mixing, and sorbic acid was added to inhibit the growth of microorganisms. At least four individuals were sampled for biological replicates at each developmental stage (Table 1). Adult beetles and locust eggs were also collected for quantifying the origins of microbial communities (Table 1).

**TABLE 1:** Detailed information of 16S rRNA gene sequencing data.

Sample	Group	Stage	Diet	Accession IDs of the data
L11	L1	First instar	Not fed	SAMN35812017
L13	L1	First instar	Not fed	SAMN35812018
L14	L1	First instar	Not fed	SAMN35812019
L15	L1	First instar	Not fed	SAMN35812020
LL21	LL2	Second instar	Locust eggs	SAMN35812021
LL22	LL2	Second instar	Locust eggs	SAMN35812022
LL23	LL2	Second instar	Locust eggs	SAMN35812023
LL24	LL2	Second instar	Locust eggs	SAMN35812024
LL25	LL2	Second instar	Locust eggs	SAMN35812025
LL31	LL3	Third instar	Locust eggs	SAMN35812026
LL32	LL3	Third instar	Locust eggs	SAMN35812027
LL33	LL3	Third instar	Locust eggs	SAMN35812028
LL34	LL3	Third instar	Locust eggs	SAMN35812029
LL35	LL3	Third instar	Locust eggs	SAMN35812030
LL41	LL4	Fourth instar	Locust eggs	SAMN35812031
LL42	LL4	Fourth instar	Locust eggs	SAMN35812032
LL43	LL4	Fourth instar	Locust eggs	SAMN35812033
LL44	LL4	Fourth instar	Locust eggs	SAMN35812034
LL45	LL4	Fourth instar	Locust eggs	SAMN35812035
AL21	AL2	Second instar	Artificial food	SAMN35812036
AL22	AL2	Second instar	Artificial food	SAMN35812037
AL23	AL2	Second instar	Artificial food	SAMN35812038
AL24	AL2	Second instar	Artificial food	SAMN35812039
AL32	AL3	Third instar	Artificial food	SAMN35812040
AL33	AL3	Third instar	Artificial food	SAMN35812041
AL34	AL3	Third instar	Artificial food	SAMN35812042
AL35	AL3	Third instar	Artificial food	SAMN35812043
AL41	AL4	Fourth instar	Artificial food	SAMN35812044
AL42	AL4	Fourth instar	Artificial food	SAMN35812045
AL44	AL4	Fourth instar	Artificial food	SAMN35812046
AL45	AL4	Fourth instar	Artificial food	SAMN35812047
AF1	~	Adult	~	Unpublished
AF2	~	Adult	~	Unpublished
AF3	~	Adult	~	Unpublished
AF4	~	Adult	~	Unpublished
AF5	~	Adult	~	Unpublished
LE1	~	Locust egg	~	Unpublished
LE2	~	Locust egg	~	Unpublished
LE3	~	Locust egg	~	Unpublished
LE4	~	Locust egg	~	Unpublished
LE5	~	Locust egg	~	Unpublished

To avoid microbial contamination in the environment, sample collection and DNA extraction were performed under sterile workbench. All tools were sterilized for 30 min at 121°C. *H. cichorii* samples were rinsed with sterile water once, soaked in 75% ethanol for 2 min, and rinsed again with sterile water to remove residual ethanol. The samples were immersed in a petri dish with PBS buffer (137 mM NaCl; 2.7 mM KCl; 10 mM Na_2_HPO_4_; 2 mM KH_2_PO_4_; and pH 7.4), and the intestines were removed under aseptic condition, immediately placed in a 2 mL centrifuge tube, frozen in liquid nitrogen, and stored in a −80°C refrigerator.

### 2.2 DNA extraction and sequencing

Total genomic DNA from samples was extracted using CTAB or SDS method. DNA concentration was determined by Nanodrop. The purity and integrity were evaluated through the 1% agarose gel electrophoresis.

According to the concentration, DNA was diluted to 1 ng/μL using sterile water. 16S rRNA genes were amplified using the specific primer with the barcode (341F: CCTACGGGNGGCWGCAG, 806R: GGACTACHVGGGTWTCTAAT). All PCR reactions were carried out in 30 μL reactions with 15 μL of High‐Fidelity PCR Master Mix (New England Biolabs); 0.2 μM of forward and reverse primers; and about 10 ng template DNA. The thermocycling conditions were as follows: 98°C pre‐degeneration for 1 min, denaturation at 98°C for 10 s, annealing at 50°C for 30 s, and extension at 72°C for 30 s, with a total of 30 cycles, followed by a final elongation step at 72°C for 5 min.

The same volume of 1× loading buffer was mixed (contained SYBR green) with PCR products and electrophoresis was operated on 2% agarose gel for detection. Samples with bright main strips between 400 and 450 bp were chosen for further experiments.

Amplicons were pooled in equal proportions and purified using TIANgel Purification Kit (TIANGEN Biotech). The purified product was used to prepare the Illumina DNA library.

Sequencing libraries were generated using TIANSeq Fast DNA Library Prep Kit (Illumina) (TIANGEN Biotech). The library quality was assessed on the Qubit@ 2.0 Fluorometer (Thermo Scientific) and Agilent Bioanalyzer 2100 system. Finally, the library was sequenced on the Illumina platform using the 2 × 250 bp paired‐end protocol. All reads have been submitted to NCBI Sequence Read Archive with SRA numbers SAMN35812017–SAMN35812047 for larvae. 16S rDNA libraries of adult guts and locust eggs will remain private until the other related manuscript has been accepted.

### 2.3 Statistical analysis

Microbiome bioinformatics was performed with QIIME 2 with slight modifications according to the official tutorials. Briefly, raw sequence data were demultiplexed using the demux plugin followed by primers cutting with the cutadapt plugin (Martin, 2011). Sequences were then quality filtered, denoised, merged, and chimera removed using the VSEARCH 2.22.1 (Rognes et al., 2016). OTUs were predicted and merged using a minimal overlap of 10 bases. Sequences with a sum of error probabilities of all bases greater than 0.01 per sequence length will be removed and excluded from further sample analysis. Chimeras were removed with the “‐‐uchime3_denovo” command of VSEARCH. There were a total of 1523 OUTs in the total dataset of 31 samples. The feature table was generated by the “‐usearch_global” command of VSEARCH (97% similarity). Species annotation was performed using QIIME2 software. The annotation database is the Greengene Database.

Alpha diversity is applied in analyzing the complexity of species diversity for a sample through four indices, including Chao1, Shannon, Richness, and ACE. Richness represents the number of species observed in each sample. Chao1 and ACE estimate the total richness. Shannon index provides information about both richness and evenness. All these indices in our samples were calculated with QIIME2 and displayed with R software. Alpha diversity measures were compared between groups using Wilcoxon's rank‐sum test and Kruskal–Wallis tests for multiple comparisons.

Beta diversity analysis was used to evaluate differences of samples in species complexity, and Beta diversity on Bray–Curtis was calculated by QIIME2 software. PERMANOVA (999 permutations, Benjamini–Hochberg corrected *p*‐values) analyses were carried out to determine the differences in community composition of bacterial microbiomes between groups based on Bray–Curtis distances data.

Principal Coordinate Analysis (PCoA) was performed to obtain principal coordinates and visualize differences of samples in complex multi‐dimensional data. A matrix of Bray–Curtis distances among samples obtained previously was transformed into a new set of orthogonal axes, where the maximum variation factor was demonstrated by the first principal coordinate, and the second maximum variation factor was demonstrated by the second principal coordinate, and so on. The three‐dimensional PCoA results were displayed using the QIIME2 package, while the two‐dimensional PCoA results were displayed using the ade package and ggplot2 package in R software (Version 3.6.2).

To evaluate the shared microbiotas between the first instar larvae and their parent, second to fourth larvae and their food, a co‐occurrence table was obtained. The taxon table was filtered using the following criterion: the co‐occurrence was assigned when a specific taxon of the first instar or second to fourth larvae microbiotas at generic and species levels was present in the microbiota of at least three adult or locust eggs samples.

The beta Nearest Taxon Index (βNTI) was calculated by comparing the observed β‐mean nearest taxon distance (βMNTD) to the mean of a null distribution of βMNTD (999 randomizations). Standard deviations were normalized using the “picante” package in R. Large absolute values (|βNTI| ≥ 2) represent deterministic processes having a dominant role in shaping microbial communities, while smaller absolute values (|βNTI| < 2) point to a stronger influence from stochastic processes instead. We then incorporated βNTI with Bray–Curtis‐based Raup–Crick indices (RCI) to quantify the ecological processes, estimating the relative strength of homogeneous selection (βNTI < −2), variable selection (βNTI > 2), homogeneous dispersal (RCI < 0.95 and |βNTI| < 2), dispersal limitation (RCI > 0.95 and |βNTI| < 2), and drift (|RCI| < 0.95 and |βNTI| < 2) in driving the composition of the microbiota.

We applied PICRUSt2 (Douglas et al., 2020), a package that contains an updated, larger database of gene families and reference genomes that is interoperable with any OTU screening and enables phenotypic prediction, for functional prediction of the gut microbiota of blister beetle larvae at different developmental stages. We used STAMP to demonstrate the differential functional pathways and for further comparison between pairwise groups. The different functional pathways between groups were predicated with a *t*‐test. LEfSe analysis was used for the quantitative analysis of biomarkers within different groups. It uses the nonparametric Kruskal–Wallis statistical test to compare all taxa at different taxonomic levels between groups and then paired Wilcoxon Rank Sum tests among subgroups. In the LEfSe analysis, *p* < .05 and LDA > 3 obtained by linear discriminant analysis were considered statistically significant.

### 2.4 Comparative analysis of carbohydrate content in locust eggs and the artificial diet

The total carbohydrate concentration of locust eggs and the artificial diet is determined by spectrophotometry based on phenol‐sulfuric acid method (Lam et al., 2021). YUAN XIN™ Total Polysaccharide Test Kit (YX‐C‐ZDT/48 samples) was used for the quantitative determination of total polysaccharides. The results of untargeted LC–MS/MS metabolomics (Zhaohui F., Unpublished) were used to compare the differences of simple carbohydrates between locust eggs and the artificial diet. Analysis of variance (ANOVA) was used for statistical test.

## 3 RESULTS

### 3.1 Overall survey of the samples

The sequenced data (31 samples) were processed for sequence quality control analysis, redundancy removal, and chimera removal (Table S1). The quality reads were binned into 1523 OTUs (Table S2). We made rarefaction curves of the data (Figure S1) to directly reflect the reasonableness of the amount of sequencing data and to indirectly reflect the richness of species in the samples. The resulting rarefaction curves showed a pattern of gradual leveling off with increasing sequencing depth. These patterns indicated that the amounts of data were reasonable since a small number of new species (OTUs) would be generated if the amount of data continued to increase.

**FIGURE 2:**
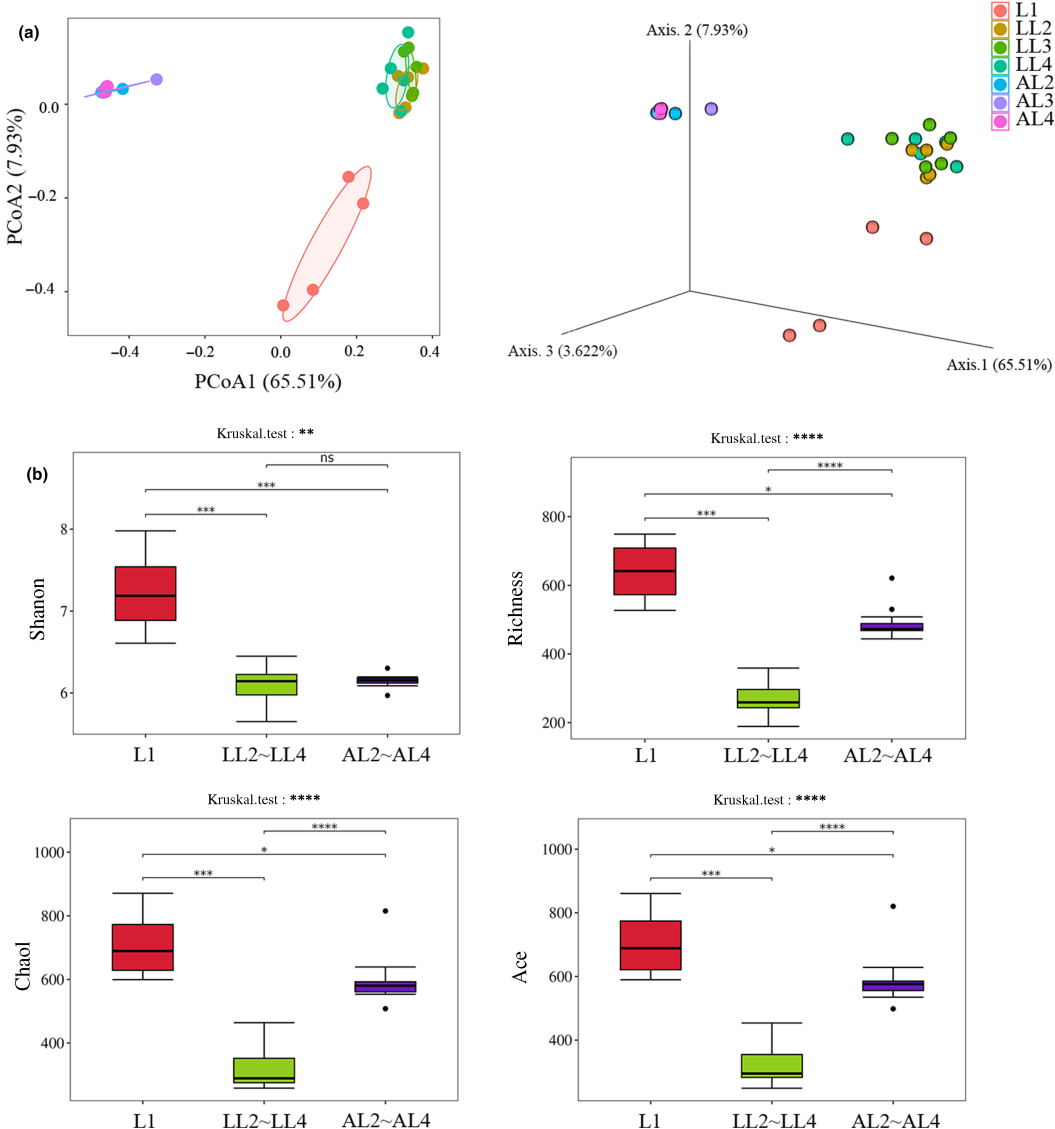
(a) Unconstrained principal coordinate analysis (PCoA) based on Bray–Curtis distance between samples. (b) The differences of α‐diversity in the three groups. The horizontal bar in the box plot represents the median, the upper and lower marginal lines represent the upper and lower quartiles (75th and 25th quartiles), and the extended lines on the margins are the extreme values in the absence of outliers, but not exceeding 1.5 times the distribution interval of the upper and lower quartiles. We use Wilcoxon's rank‐sum test to compare the differences between groups and use Kruskal–Wallis tests for multiple comparisons. L1: First instar larvae; LL2~LL4: second to fourth instar larvae fed locust eggs; AL2~AL4: second to fourth instar larvae fed artificial diet.

We further performed unconstrained principal coordinate analysis (PCoA) on the Bray–Curtis distance between samples, and the results of 2D and 3D PCoA showed that the gut microbiota was roughly divided into three clusters on the first axis, which indicated that the structure of the gut microbiota of the first instar larvae (L1 group), second to fourth instar larvae fed with locust eggs (LL2~LL4 group), and second to fourth instar larvae fed with artificial food during were significantly different (AL2~AL4 group) (Figure 2a and Table S3). Pairwise permanova results also showed significant differences between larvae on different diets (Table S4). When comparing the within‐sample bacterial diversity (α‐diversity), marked differences were observed between the L1 and LL2~LL4 groups or the L1 and AL2~AL4 groups based on the Shannon index. We found that there were also significant differences in the α‐diversity of gut microbiota between the L1 group, LL2~LL4 group, and AL2~AL4 group based on the richness, chao1, and ace index, with the diversity higher in the L1 group than in the LL2~LL4 group and AL2~AL4 group (Figure 2b). These findings indicated distinct variations in the gut microbiota of the first instar larvae and the second to the fourth instar larvae fed by different diets. Furthermore, the gut microbiota in first instar larvae displayed greater complexity compared to that of other larvae.

### 3.2 Differences in gut microbiota among groups at different taxonomic levels

**FIGURE 3:**
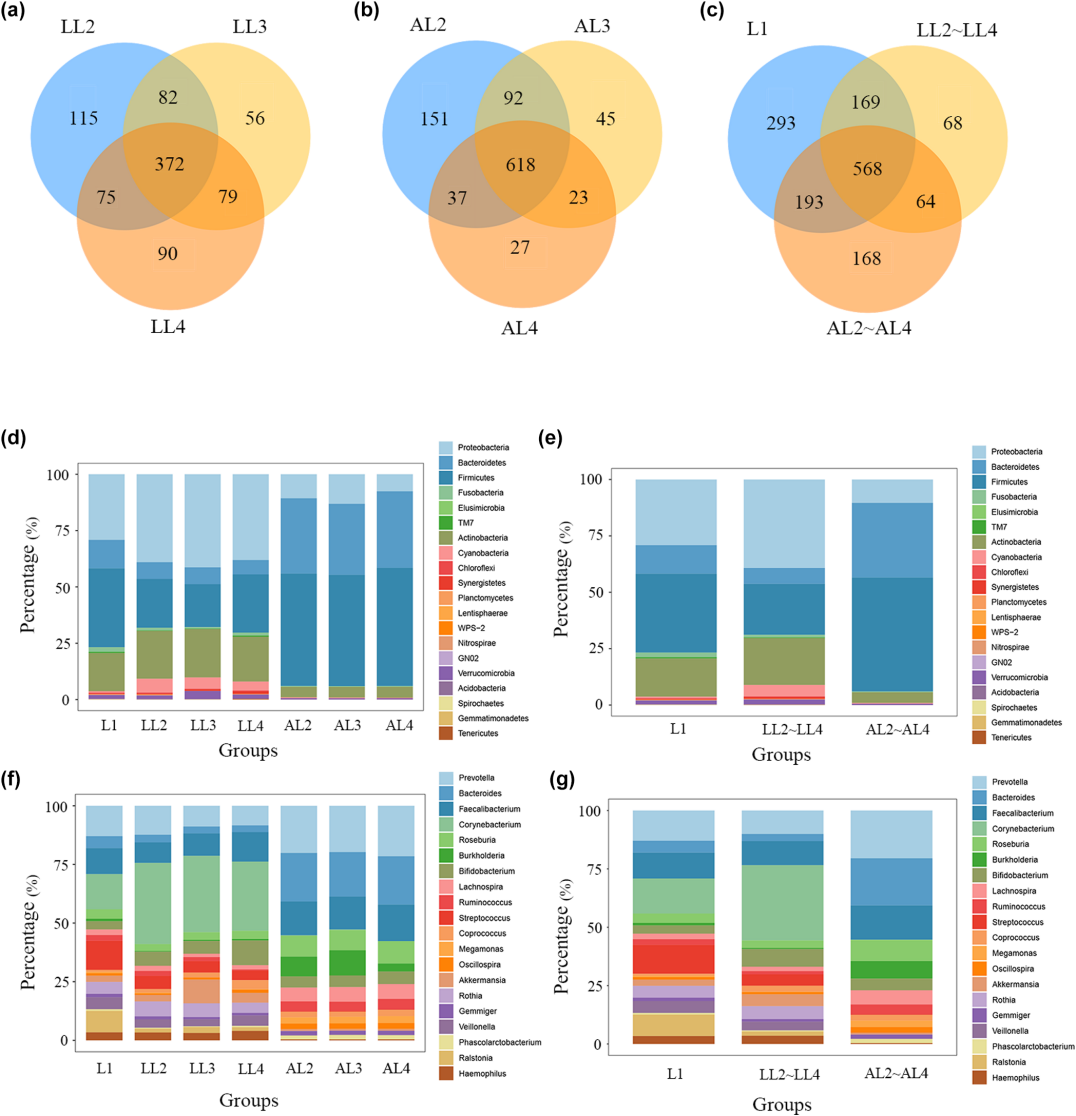
Differences of gut microbiota among groups at the OTU level and phylum/genus level. (a) Venn diagram showing the OTUs shared among the LL2, LL3, and LL4 samples. (b) Venn diagram showing the OTUs shared among the AL2, AL3, and AL4 samples. (c) Venn diagram showing the OTUs shared among the L1, LL2~LL4, and AL2~AL4 samples. (d) Histogram of the relative abundance of the 20 most abundant phyla in the gut microbiota of the L1, LL2, LL3, LL4, AL2, AL3, and AL4 samples. (e) Histogram of the relative abundance of the 20 most abundant phyla in the gut microbiota of the L1, LL2~LL4, and AL2~AL4 groups. (f) Histogram of the relative abundance of the 20 most abundant genera in the gut microbiota of the L1, LL2, LL3, LL4, AL2, AL3, and AL4 samples. (g) Histogram of the relative abundance of the 20 most abundant genera in the gut microbiota of the L1, LL2~LL4, and AL2~AL4 groups. L1: First instar larvae; LL2~LL4: second to fourth instar larvae fed locust eggs; AL2~AL4: second to fourth instar larvae fed artificial diet.

We first analyzed the gut microbial richness at the OTU level (Figure 3a–c). There were 1223 OTUs in the first instar larvae, 869 OTUs in the second to fourth instar larvae fed with locust eggs, and 993 OTUs in the second to fourth instar larvae fed with the artificial diet. Among them, 372 and 618 OTUs were common to second to fourth instar larvae fed locust eggs and second to fourth instar larvae fed artificial diet, separately (Figure 3a,b). We found that the number of OTUs shared among L1, LL2~LL4, and AL2~AL4 groups was 568, accounting for 46.4% OTUs of the L1 group, 65.4% OTUs of the LL2~LL4 group, and 57.2% of the AL2~AL4 group. The number of OTUs unique to the gut microbiota of L1, LL2~LL4, and AL2~AL4 groups was 293, 68, and 168. The L1 group had a more abundant gut microbiota than the LL2~LL4 and AL2~AL4 groups (Figure 3c).

The taxon of each OUT is shown in Table S5. We compared the composition of gut bacteria in first, second, third, and fourth instar larvae to each other, and the bacterial flora contents were highly similar among second, third, and fourth instar larvae at both phylum and genus levels (Figure 3d,f), so we combined second to fourth instar larvae together in the following analysis. At the phylum level, the major flora of the insect gut microbiota in all groups were Firmicutes, Bacteroidetes, Actinobacteria, and Proteobacteria (Figure 3e). The major bacterial flora contents were similar among these three groups, except that the AL2~AL4 group contained a relatively higher proportion of the bacterial composition of Bacteroidetes (33.1%). At the genus level, the gut microbiota of the L1 group was mainly composed of *Corynebacterium*, *Prevotella*, and *Streptococcus*, with relative abundances of 15.0%, 12.9%, and 12.4%, respectively. *Corynebacterium*, *Faecalibacterium*, and *Prevotella* were the relatively more abundant genera in the LL2~LL4 group, with a relative abundance of 32.2%, 10.4%, and 10.0%, respectively. AL2~AL4 group was mainly composed of *Prevotella*, *Bacteroides*, and *Faecalibacterium*, with relative abundances of 20.4%, 20.2%, and 14.7%, respectively (Figure 3g). This indicated that there was a large difference in the relative abundance of species of gut microbiota among groups at the genus level.

Adult beetles and locust eggs metagenomic libraries were generated to determine the extent to which the bacterial species of the insect gut could reflect the environmental and food bacterial communities. The top three genera found in adults were *Prevotella*, *Bacteroides*, and *Faecalibacterium*, but *Corynebacterium*, *Streptococcus*, and *Prevotella* in the L1 group (Figure S2a,b). A total of 38 genera and 36 species annotated in gut microbes of first instar larvae were also found in adult samples (Table S6). Many bacterial classes described in the guts of LL2~LL4 were observed in low proportion in locust eggs, such as *Corynebacterium*, *Faecalibacterium*, and *Prevotella*. *Streptococcus* found in locust eggs was greater than 60%. Nonetheless, the abundance of these bacteria in the LL2~LL4 was very low (Figure S2c,d). Only 10 species and 22 genera were in co‐occurrence in the LL2~LL4 group and locust eggs (Table S7). Overall, the overlap between the bacterial species found in the guts of the LL2~LL4 group and those detected in the locust eggs was minimal.

### 3.3 Mechanisms of microbial community assembly

**FIGURE 4:**
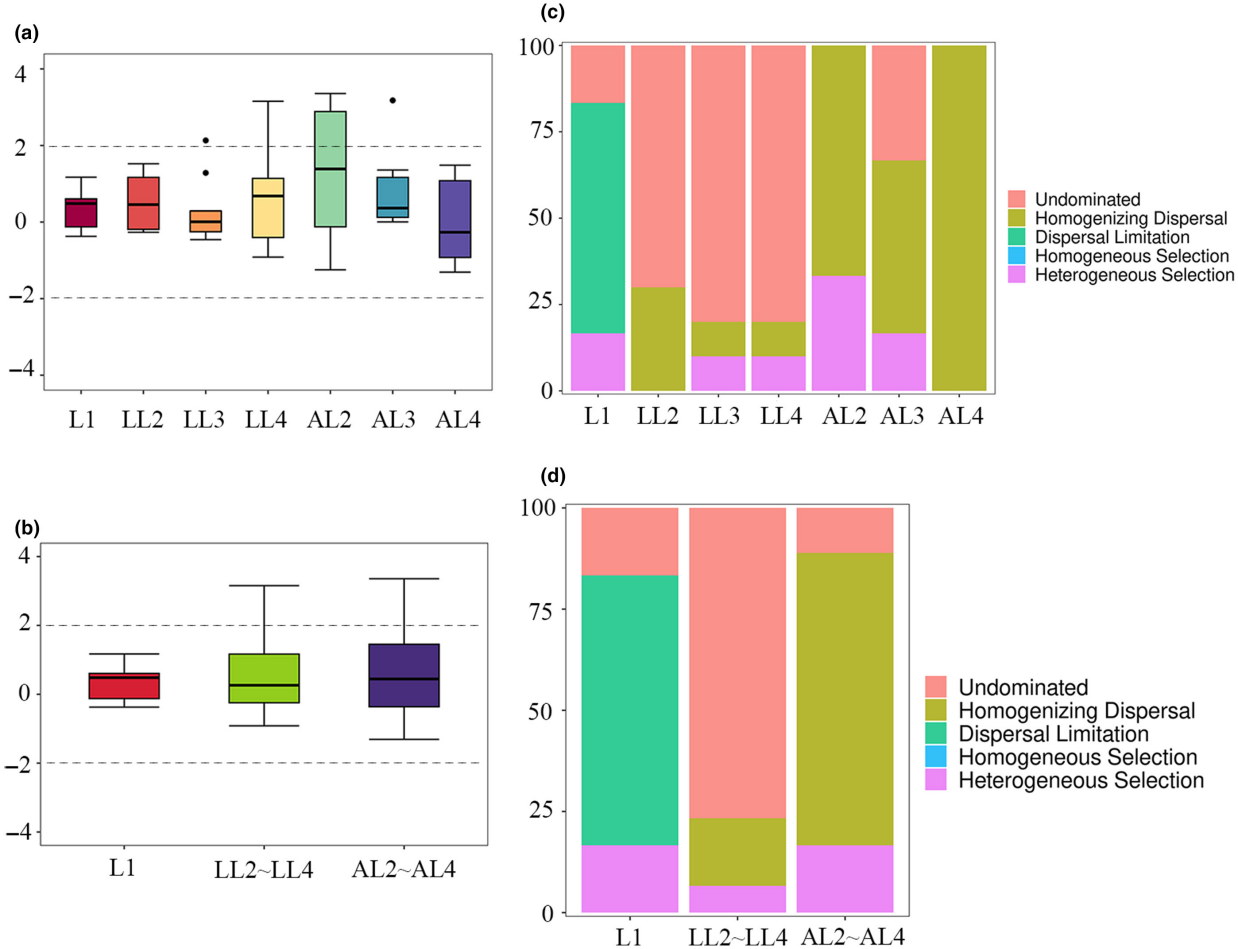
Mechanisms of microbial community assembly in different groups. Box and whisker plots of contributions of deterministic (|βNTI| ≥ 2) and stochastic processes (|βNTI| < 2) on microbial community assembly in each group (a and b). The relative contributions of ecological processes in driving the assembly in each group (c and d). L1: First instar larvae; LL2~LL4: second to fourth instar larvae fed locust eggs; AL2~AL4: second to fourth instar larvae fed artificial diet.

The relative contribution of different ecological processes in shaping microbiota assembly in different instars and diets was quantified with null model analyses (Figure 4). Results showed that microbial community assembly in each of the groups was primarily driven by stochastic processes (−2 < βNTI < 2) (Figure 4a,b). In the L1 group, dispersal limitation was the top factor driving microbial community. Undominated had a higher relative contribution to the assembly of microbial community in the gut of the LL2~LL4 group. On the other hand, the relative influence of homogenizing dispersal was higher for communities in the AL2~AL4 group (Figure 4c,d). Collectively, these results demonstrate that stochastic processes, including drift and dispersal, dominantly drive the community assembly of the gut microbiota, and that their relative influence is dependent on the taxonomy of the host.

### 3.4 Functional prediction of the gut microbial communities

**FIGURE 5:**
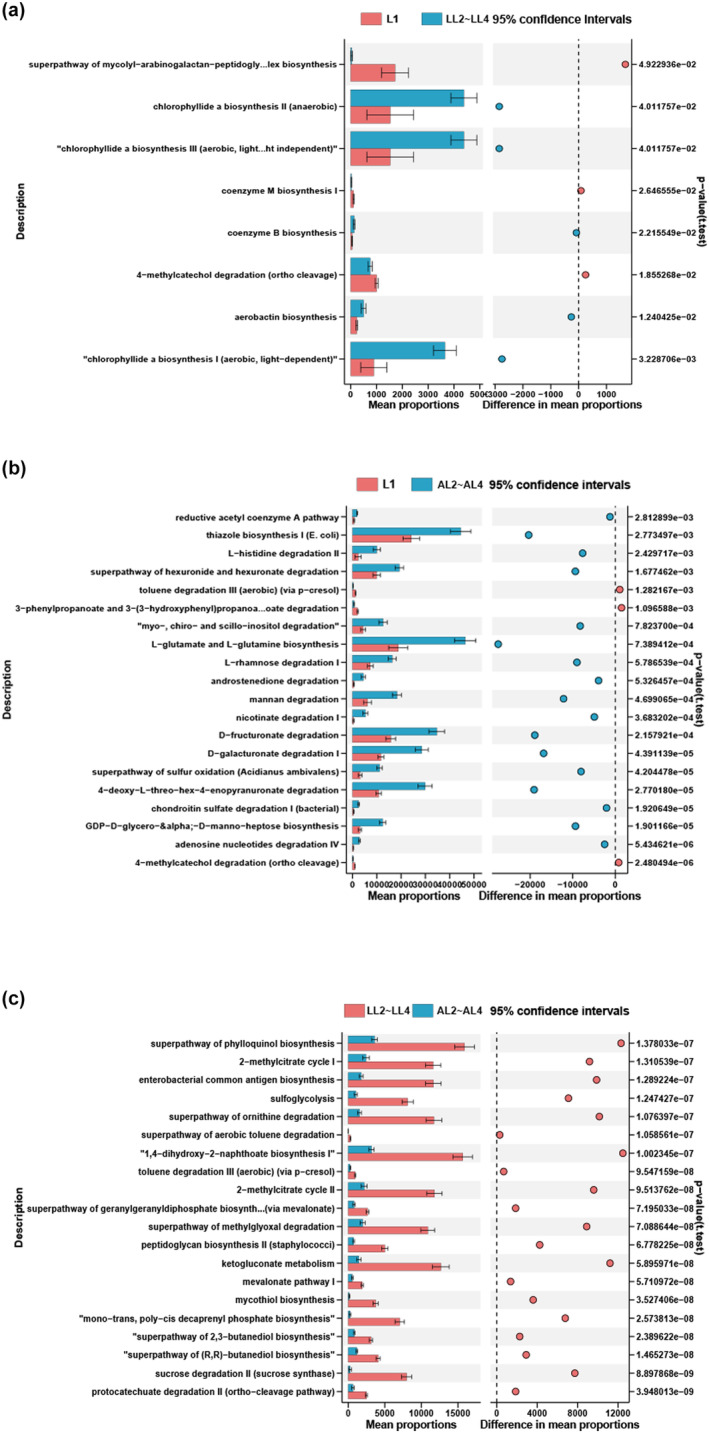
Differential functional pathways of gut microbes in three groups predicted by PICRUSt2. (a) Differential functional pathways between L1 and LL2~LL4 groups. (b) Differential functional pathways between L1 and AL2~AL4 groups (the top 20 significant items are shown). (c) Differential functional pathways between LL2~LL4 and AL2~AL4 groups (The top 20 significant items are shown). L1: First instar larvae; LL2~LL4: second to fourth instar larvae fed locust eggs; AL2~AL4: second to fourth instar larvae fed artificial diet.

A total of eight functional pathways were identified as significant differences between the L1 and LL2~LL4 groups (Figure 5a and Table S8). Notably, in the LL2~LL4 group, pathways related to chlorophyllide biosynthesis, such as chlorophyllide a biosynthesis I (aerobic, light‐dependent), chlorophyllide a biosynthesis III (aerobic, light independent), and chlorophyllide a biosynthesis II (anaerobic), were increased, while 4‐methylcatechol degradation (ortho cleavage), coenzyme M biosynthesis I, and superpathway of mycolyl‐arabinogalactan‐peptidoglycan complex biosynthesis were decreased. In the AL2~AL4 group, pathways related to substrates degradation such as carbohydrate degradation, inorganic nutrient metabolism, fatty acid and lipid degradation, and amino acid degradation were higher, compared to that in the L1 group (Figure 5b and Table S9). Specifically, the extended error bar plot showed that the mean proportion (%) of the top 20 significant items, which were involved in both substrate degradation and biosynthesis in the gut of the LL2~LL4 group, were all significantly higher than that in the AL2~AL4 group (Figure 5c). However, many terms related to polysaccharide utilization were significantly higher in the AL2~AL4, including chitin derivatives degradation, mannan degradation, chondroitin sulfate degradation I (bacterial), glycogen degradation I (bacterial), and starch degradation V (Table S10).

### 3.5 Prediction of biomarker taxa by LEfSe based on species compositions

**FIGURE 6:**
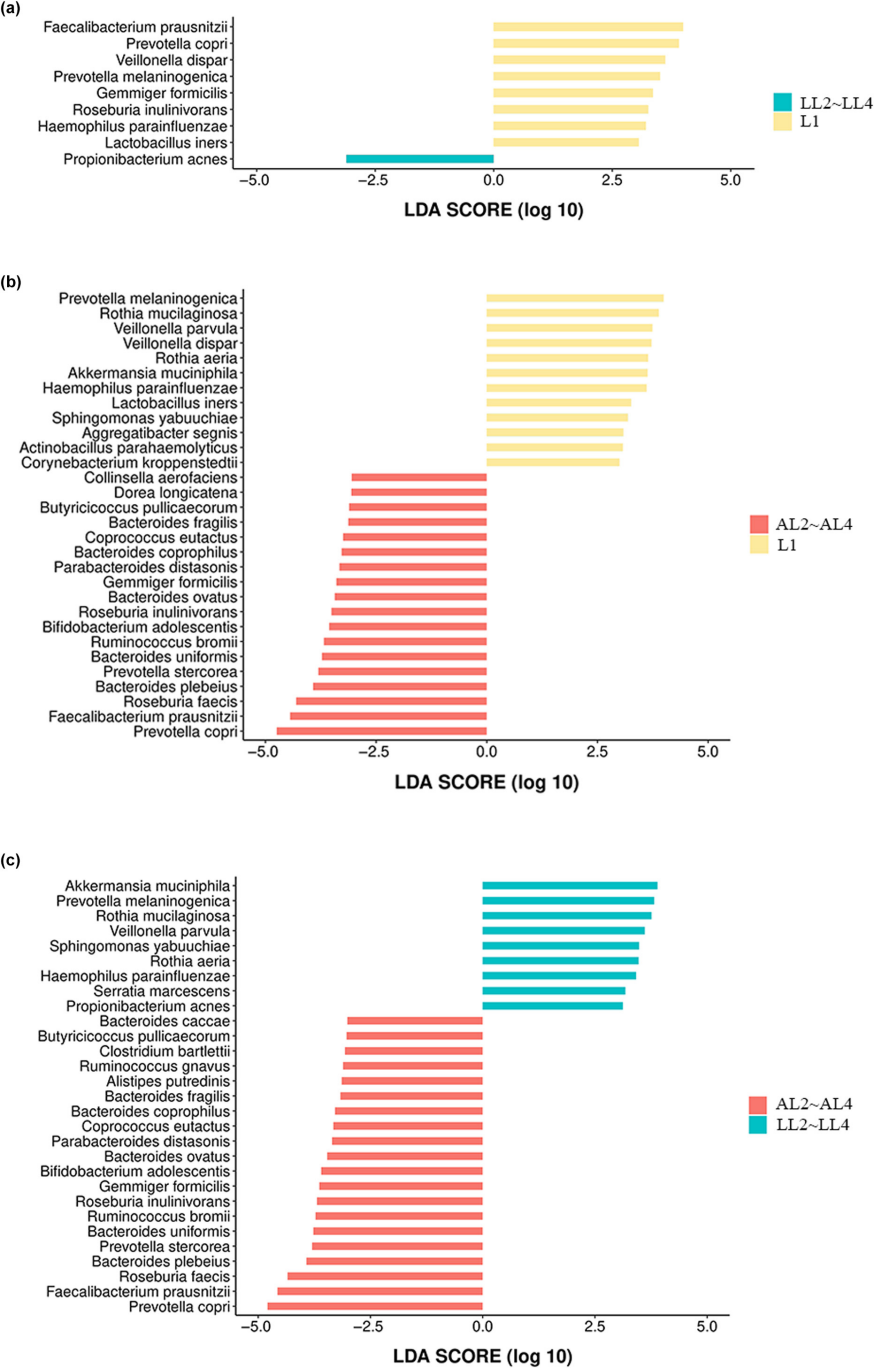
LEfSe analysis between different groups. (a) The different microbes between the L1 and LL2 ~ LL4 groups by LEfSe. (b) The different microbes between the L1 and AL2 ~ AL4 groups by LEfSe. (c) The different microbes between the LL2 ~ LL4 and AL2 ~ AL4 groups by LEfSe. L1: First instar larvae; LL2 ~ LL4: second to fourth instar larvae fed locust eggs; AL2 ~ AL4: second to fourth instar larvae fed artificial diet.

Significant biomarker taxa between each group were shown in Tables [Link], [Link], [Link]. Significant changes were observed in nine microbe species between the L1 and LL2~LL4 groups (Figure 6a). *Propionibacterium acnes* was significantly enriched in the LL2~LL4 group, while others were significantly enriched in the L1 group. Thirty microbe species were significantly different between the L1 and AL2–AL4 groups (Figure 6b). Eight species related to polysaccharide metabolism including *Prevotella copri*, *Prevotella stercorea*, *Faecalibacterium prausnitzii*, *Ruminococcus bromii*, *Collinsella aerofaciens*, *Bacteroides ovatus*, *Bacteroides uniformis*, and *Parabacteroides distasonis* were significantly enriched in the AL2~AL4 group. Significant changes were observed in 29 microbe species between the LL2~LL4 and AL2~AL4 groups (Figure 6c). Nine species were significantly enriched in the LL2~LL4 group, while the other 20 species were significantly enriched in the AL2~AL4 group including *Prevotella copri*, *Prevotella stercorea*, *Faecalibacterium prausnitzii*, *Ruminococcus bromii*, *Bacteroides ovatus*, *Bacteroides uniformis*, *Parabacteroides distasonis*, and so on.

## 4 DISCUSSION

In our study, the gut microbiota in first instar larvae displayed greater complexity compared to that of the other larvae fed by different diets (Figure 2b). According to our results, common soil microbes, including *Actinomyces*, *Kaistobacter*, and *Hylemonella* genera, were indeed found in first instar larvae (Table S11). Moreover, to detect whether the first instar larvae gut microbes came from the adult samples, gut microbiomes of five female adults were examined by 16S rDNA amplicon sequencing. The 38 genera and 36 species annotated in gut microbes of first instar larvae were also found in adult samples (Table S6). Insects can adopt two non‐mutually exclusive strategies to acquire microorganisms throughout development; one is from direct contact with their environment and the other is from their parents (Cheutin et al., 2024). The first instar larvae of *H. cichorii* may employ both strategies, thus enriching the diversity of intestinal microbes.

We found that only a tiny minority of gut microbiota within LL2~LL4 samples might draw from their food, indicating that the diet was not a primary mechanism of microbiota acquisition for *H. cichorii* larva. For example, *Propionibacterium acnes*, which plays an important role in producing propionate and vitamin B12 in the gut of insects (Jang et al., 2022; Wang et al., 2011), was enriched in the locust‐fed larvae but was nonexistent in locust eggs. Dietary components, environmental location, and gut morphology act as selective agents sculpting the composition of intestinal microbiota (Björk et al., 2019; Zhu et al., 2022). This likely led to a mutualistic or symbiotic association between *P. acnes* and locust‐fed larvae in the gut.

### 4.1 The presence of potential gut microbiota that influences the diet of first instar larvae

Our previous research has shown that the feeding specialization of *H. cichorii* larvae on locust eggs gradually decreases with individual development. First instar larvae can hardly develop by being fed on the artificial diet, upon which the older instars grow and develop well (Fu et al., 2023). As shown in Figure 6b, eight biomarker taxa that were abundant in AL2~AL4 groups were involved in carbohydrate metabolism processes, including *Prevotella copri*, *Prevotella stercorea*, *Faecalibacterium prausnitzii*, *Ruminococcus bromii*, *Collinsella aerofaciens*, *Bacteroides ovatus*, *Bacteroides uniformis*, and *Parabacteroides distasonis*. They have complex enzymatic machinery to utilize complex polysaccharides including arabinogalactan, xylan, fiber, d‐glucosamine, N‐acetyl‐d‐glucosamine, and starch as nutrients (Bag et al., 2017; Brown et al., 2023; Ezeji et al., 2021; Leylabadlo et al., 2020; López‐Almela et al., 2021; Prasoodanan et al., 2021; Rangarajan et al., 2022; Yang et al., 2021). We compared the concentrations of total carbohydrates and total polysaccharides in locust eggs and the artificial diet (Table S14). The artificial diet we employed contains a higher amount of carbohydrates and polysaccharides than locust eggs. Yeast powder and honey were the main ingredients in the artificial diet. Yeast contains yeast polysaccharides, and the main efficient ingredients are β‐glucan and mannan. Nectar, the raw material of honey, contains polysaccharides including cellulose, hemicellulose, and pectin. These potential energy sources could be degraded and fermented through microbial enzymatic activity, resulting in short‐chain fatty acids available to hosts (Zheng et al., 2019). Therefore, we speculated the artificial diet failed to support the growth of first instar larvae probably because of excessive polysaccharides content in the artificial food, which was difficult for first instar larvae to digest and utilize. The composition and metabolism of the gut microbiota are influenced by dietary structure, particularly the type and balance of the primary dietary macronutrients (carbohydrates, proteins, and fats) (Douglas, 2015). If an excess of one nutrient is present, it would give rise to deficiencies in other components present in concentrations and limit the growth and development of the host (Zhu et al., 2022). It is unclear if and how carbohydrate and/or polysaccharide complexity of diets may encourage host‐microbe associations in blister beetle larvae but warrants further exploration. This would help us understand the nutritional requirements of blister beetle larvae and guide the optimization of the artificial diet.

### 4.2 Significant differences in the structure of gut microbiota between artificial diet‐fed and locust eggs‐fed larvae

Bacterial population dynamics were strongly influenced by the diet the insect consumed (Yun et al., 2014). There is no significant difference in the gut microbial structure among the second to the fourth instar larvae under the same rearing conditions, but the gut bacterial populations responded differently to diets of locust eggs or artificial diets. At the OTU level, the gut microbiota is more abundant in the AL2~AL4 group (993 OTUs) than in the LL2~LL4 group (869 OTUs). At the genus level (Figure 3g), there are also large differences in gut microbiota between the two groups, with *Corynebacterium* and *Faecalibacterium* being relatively more abundant (%) in the LL2~LL4 group, while *Prevotella* and *Bacteroides* being relatively more abundant in the AL2~AL4 group. *Corynebacterium* is a genus of Gram‐positive bacteria and most are aerobic. It is involved in hydrocarbon degradation (it is critical for the breakdown and elimination of environmental toxins) (Azam et al., 2023). Members of the genus *Faecalibacterium* are considered a commensal microorganism, ubiquitous within the gastrointestinal tracts of animals and humans (Tap et al., 2009). They play a crucial role in carbohydrate utilization and energy production (Martín et al., 2023). Most bacteria of *Bacteroides* possess enzymes that hydrolyze polysaccharides, which can convert complex polysaccharides into simple nutrients that can be used by the host (Xu et al., 2007), and also have extensive carbohydrate utilization activities (Flint, 2011). *Prevotella* species are often linked with reduced visceral fat and improved glucose metabolism (Asnicar et al., 2021; de León et al., 2020; Kovatcheva‐Datchary et al., 2015; Salazar‐Rivera et al., 2024). These results suggest that microbial structures vary widely between artificial diet‐fed and locust eggs‐fed larvae. The comparative analysis showed that the content of carbohydrates in locust eggs and artificial diet were different. The content of sucrose in locust eggs was higher than that in artificial diet, while the content of polysaccharides and most of the simple carbohydrates (including glucose, maltose, fructose, and lactose) in artificial diet was higher than that in locust eggs (Tables S14 and S15). The difference in gut microbial composition may be related to the utilization of different carbs by larvae. However, since most of the effect‐mechanism relationships have not been fully elucidated, microbial‐host interactions still need to be studied, and the mechanisms by which nutrition utilization is influenced by gut microbes require further investigation.

Functional prediction of microbial communities also showed similar results. The results of differential functional pathways predicted by PICRUSt2 show that the mean proportion (%) of functions involved in metabolism in the gut of the LL2~LL4 group were all significantly higher than that in the AL2~AL4 group. To screen out the gut microbiota that may play an important role in the nutrition metabolism of different diet samples, we apply LEfSe analysis to classify the hosts according to the gut microbial biomarker taxa. Among the biomarker taxa of high importance, *Akkermansia muciniphila*, *Rothia mucilaginosa*, and *Rothia aeria* have higher relative abundances in the gut microbiota of the LL2~LL4 group. *A. muciniphila* is regarded as a promising “next‐generation beneficial microbe” for metabolic disease prevention or therapy owing to its various properties, including producing short‐chain fatty acids, improving intestinal integrity, and reducing endotoxemia through inhibiting the translocation of lipopolysaccharide from the intestine to circulation (Yan et al., 2021). *Rothia mucilaginosa* and *Rothia aeria* are members of the *Rothia* genus. *Rothia* species has an extensive number of CAZy, which are associated with (exo) polysaccharides production and catabolism of carbohydrate sources (Oliveira et al., 2022). These results further suggest that these gut microbial communities in the larvae fed with locust eggs may have functions in substance metabolism and degradation, helping the hosts adapt to the environment and contributing to the production of active ingredients. Results also showed that the relative abundance of polysaccharide utilization‐related bacteria was significantly higher in the AL2~AL4 group compared to the LL2~LL4 group including *Prevotella copri*, *Faecalibacterium prausnitzii*, *Ruminococcus bromii*, *Bacteroides ovatus*, *Bacteroides uniformis*, and *Parabacteroides distasonis*, which showed the same result when compared to the L1 group. The difference in nutrient composition between artificial diet and natural food would be the reason for the emergence of different types of bacteria.

## 5 CONCLUSION

In this study, we completed a comparative analysis of the composition and diversity of larvae gut microbes through different feeding in *H. cichorii* using 16S rDNA amplicon sequencing. There is no significant difference in the gut microbial structure among the second to the fourth instar larvae under the same rearing conditions, but the gut microbial structure of the first instar larvae was significantly different from the second to the fourth instar larvae fed by different diets. Bacteria associated with polysaccharide utilization are relatively barren in first instar larvae. Comparing the carbohydrate content between the artificial diet and locust eggs, we speculate that an excessive amount of carbohydrates, especially polysaccharides in the artificial diet, may be detrimental to the growth and development of first instar larvae. Artificial feed affected the composition and structure of the gut microbiota of the second to the fourth instar larvae. The difference in gut microbial composition may be related to the utilization of different nutrients in diet by larvae. In the following research, we should optimize the artificial diet formula by comparing the nutritional composition between the artificial diet and locust eggs. In particular, the content of polysaccharides and simple carbohydrates in artificial diet should be reduced to achieve the feeding of the first instar larvae.

## AUTHOR CONTRIBUTIONS

**Jinnan Ma:** Formal analysis (equal); methodology (equal); software (equal); writing – original draft (equal). **Zhaohui Fu:** Investigation (equal); visualization (equal); writing – original draft (equal). **Xin Yang:** Formal analysis (equal); validation (equal). **Wenqin Ming:** Data curation (equal); software (equal). **Xuhao Song:** Resources (equal); writing – review and editing (equal). **Chao Du:** Conceptualization (equal); funding acquisition (equal); methodology (equal); project administration (equal); supervision (equal); writing – review and editing (equal).

## CONFLICT OF INTEREST STATEMENT

None declared.

## Supporting information


Figure S1.

Figure S2.

Table S1.

Table S2.

Table S3.

Table S4.

Table S5.

Table S6.

Table S7.

Table S8.

Table S9.

Table S10.

Table S11.

Table S12.

Table S13.

Table S14.

Table S15.

Data S1:

## Data Availability

All reads have been submitted to NCBI Sequence Read Archive with SRA numbers SAMN35812017–SAMN35812047.
