# Supplementary figures and images for: Gut microbial changes in a specialist blister beetle larvae and their nutritional metabolic characteristics
Source: Ecol Evol. 2024 Aug 22;14(8):e70184. doi: 10.1002/ece3.70184 (PMC11341433; doi:10.1002/ece3.70184)

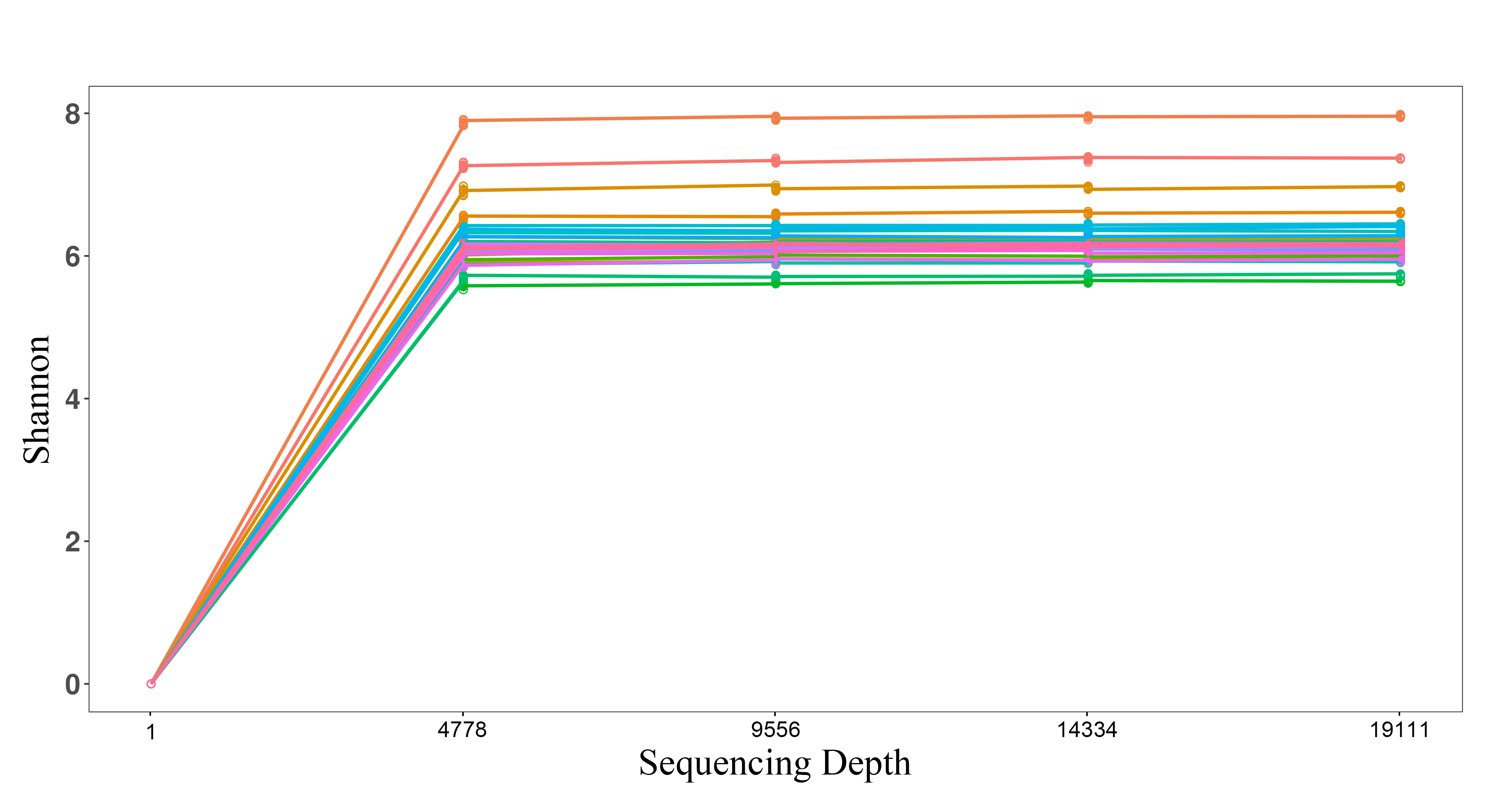


**Supplementary Figure 1** Data rarefaction curves of gut microbiota in groups.

Supplement: Supplementary file 1 — Figure S1. [file ECE3-14-e70184-s007.docx]
