# Supplementary material for: Gut microbial changes in a specialist blister beetle larvae and their nutritional metabolic characteristics
Source: Ecol Evol. 2024 Aug 22;14(8):e70184. doi: 10.1002/ece3.70184 (PMC11341433; doi:10.1002/ece3.70184)
Supplement: Supplementary file 2 — Figure S2. [file ECE3-14-e70184-s017.docx]

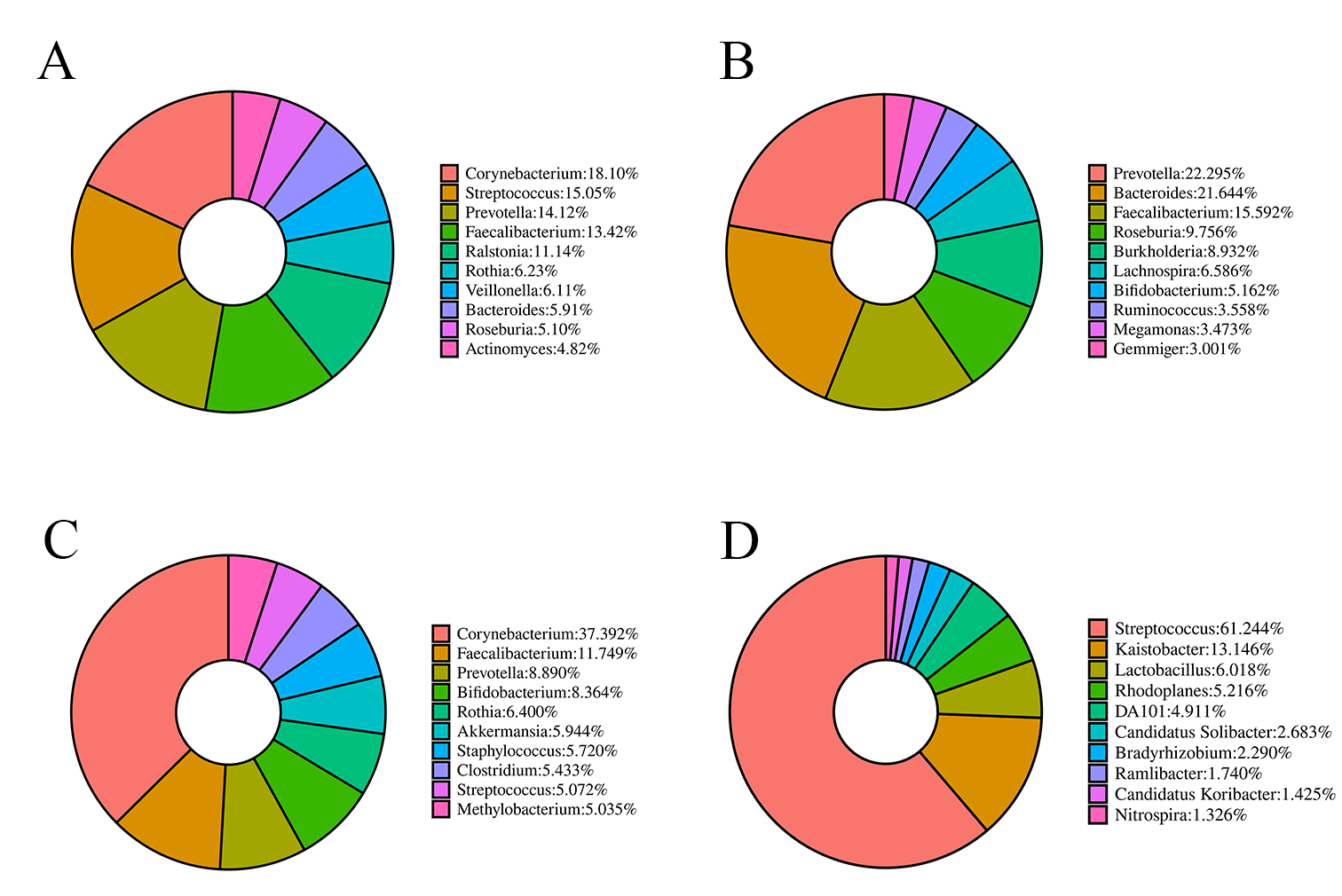


**Supplementary Figure 2** Relative abundance (by percentage) of bacterial classes found in the guts of L1 group (A), adult samples (B), LL2~LL4 group (C), and locust eggs (D). L1: first instar larvae; LL2~LL4: 2nd to 4th instar larvae fed locust eggs.
